# Developing and testing a brief clinic‐based lung cancer screening decision aid for primary care settings

**DOI:** 10.1111/hex.12675

**Published:** 2018-02-23

**Authors:** Karen Kane McDonnell, Scott M. Strayer, Erica Sercy, Callie Campbell, Daniela B. Friedman, Kathleen B. Cartmell, Jan M. Eberth

**Affiliations:** ^1^ College of Nursing University of South Carolina Columbia SC USA; ^2^ Department of Family and Preventive Medicine School of Medicine University of South Carolina Columbia SC USA; ^3^ Department of Epidemiology and Biostatistics Arnold School of Public Health University of South Carolina Columbia SC USA; ^4^ Statewide Cancer Prevention and Control Program Arnold School of Public Health University of South Carolina Columbia SC USA; ^5^ Department of Health Promotion, Education, and Behavior Arnold School of Public Health University of South Carolina Columbia SC USA; ^6^ College of Nursing Medical University of South Carolina Columbia SC USA

**Keywords:** decision support, early detection of cancer, lung neoplasms, patient preferences, primary care providers

## Abstract

**Background:**

Cancer screening‐related decisions require patients to evaluate complex medical information in short time frames, often with primary care providers (PCPs) they do not know. PCPs play an essential role in facilitating comprehensive shared decision making (SDM).

**Objective:**

To develop and test a decision aid (DA) and SDM strategy for PCPs and high‐risk patients.

**Design:**

The DA was tested with 20 dyads. Each dyad consisted of one PCP and one patient eligible for screening. A prospective, one‐group, mixed‐method study design measured fidelity, patient values, screening intention, acceptability and satisfaction.

**Results:**

Four PCPs and 20 patients were recruited from an urban academic medical centre. Most patients were female (n = 14, 70%), most had completed high school (n = 15, 75%), and their average age was 65 years old. Half were African American. Patients and PCPs rated the DA as helpful, easy to read and use and acceptable in terms of time frame (observed *t* = 11.6 minutes, SD 2.7). Most patients (n = 16, 80%) indicated their intent to be screened. PCPs recommended screening for most patients (n = 17, 85%).

**Conclusions:**

Evidence supports the value of lung cancer screening with LDCT for select high‐risk patients. Guidelines endorse engaging patients and their PCPs in SDM discussions. Our findings suggest that using a brief, interactive, plain‐language, culturally sensitive, theory‐based DA and SDM strategy is feasible, acceptable and may be essential to effectively translate and sustain the adoption of LDCT screening recommendations into the clinic setting.

## 1 INTRODUCTION

Cancer screening‐related decisions can require patients to evaluate complex medical information in short time frames, often with primary care providers (PCPs) they do not know. Intense emotions about the possibility of a cancer diagnosis may create anxiety and affect decision making. In the United States (U.S.), the Centers for Medicare and Medicaid Services (CMS) decision to include low‐dose computed tomography (LDCT) for lung cancer screening as a reimbursable service with the requirement that a “counselling and shared decision making” (SDM) visit with a PCP precede the screening is evidence of the anticipated complexity of making this decision and the importance of weighing its risks and benefits.^1^, ^2^ PCPs play an essential role in facilitating comprehensive SDM.^3^, ^4^ SDM was endorsed by the U.S. Preventive Services Task Force in its Screening for Lung Cancer Recommendation Statement and is viewed as a way to minimize concerns related to implementation variances.^5^

SDM is a collaborative communication strategy that allows patients and their PCPs to make health‐care decisions together, taking into account the best clinical evidence available as well as the patient's values and preferences. SDM incorporates the patient's voice in health‐care decisions and is described as the pinnacle of patient‐centred care.^6^, ^7^, ^8^ The principles of self‐determination (the freedom of patients to make their own choices) and relational autonomy (an understanding that decisions are made in the context of interpersonal relationships and mutual dependencies) are important precursors to the integration of SDM into clinical practice. Even though the importance of these principles has been well documented, there is a lack of practical guidance about how to implement SDM between PCPs and patients in routine clinical settings where time is limited and there are often competing priorities.^4^, ^9^, ^10^, ^11^

Elwyn and colleagues^4^ describe a three‐step SDM model that is practical, is easy to remember and can act as a guide to skill development: (i) introducing choice; (ii) describing options, often by integrating the use of decision support tools or aids; and (iii) helping patients explore preferences and make decisions. This model involves deliberation, with the understanding that decisions should be influenced by respecting “what matters most” to patients as individuals and that the decision should also rely on patients developing informed preferences.^4^ SDM emphasizes building a good relationship in the clinical encounter so that information is shared and patients are encouraged and supported to express their preferences during the decision‐making process.^4^, ^12^, ^13^

Evidence strongly indicates that decision support tools or aids lead to improved knowledge, reduced decisional conflict and decisions that are compatible with the patient's value system.^14^, ^15^, ^16^, ^17^, ^18^, ^19^ There are several published screening decision aids (DAs) for patients at risk for lung cancer; however, they are not all interactive, written in plain language, sensitive to diverse cultures or designed to be used in brief clinical encounters (see Table 1).^16^, ^17^, ^18^, ^19^, ^20^, ^21^, ^22^, ^23^ The purpose of this pilot study was to test the feasibility and acceptability of implementing a brief, clinic‐based DA written with lower readability and enhanced cultural sensitivity and a SDM strategy developed for PCPs to use in clinical settings as they discuss the pros and cons of lung cancer screening with their high‐risk patients.

**Table 1:** Decision aids evaluated against international standards

Title, developer, access	Audience, format(s), costs	IPDAS^a^^,^ b criteria (readability^c^)
“Is Lung Cancer Screening Right for Me?” Agency for Healthcare Research and Quality (AHRQ) http://effectivehealthcare.ahrq.gov/tools-and-resources/patient-decision-aids/lung-cancer-screening/decisionmaking-tool 19 (print versions can be ordered by calling the AHRQ Publications Clearinghouse toll‐free at 800‐358‐9295 or emailing AHRQPubs@ahrq.hhs.gov)	Adult patients and health‐care providers (HCPs) English/Spanish versions (of some components), part of a multicomponent resource, paper version available, can be downloaded and printed or ordered by telephone Free	Defined as a DA: 7 of 7 criteria Lowers the risk of bias: 9 of 9 criteria Other screening issues: 4 of 4 criteria Other quality issues: 10 of 13 criteria (Does not report readability levels: Average readability determined = Grade level 11.5)
Option Grid™ EBSCO Health, EBSCO Information Services http://optiongrid.org 16	Adult patients at high risk for lung cancer are encouraged to share the one‐page DA (taken from Option Grid's library of DAs on a variety of health topics) with HCPs English, Web‐based, paper version available plus PDF and online interactive versions that can be downloaded and printed Free	Defined as a DA: 7 of 7 criteria Lowers the risk of bias: 6 of 9 criteria Other screening issues: 4 of 4 criteria Other quality issues: 6 of 13 criteria (Does not report readability levels: Average readability determined = Grade level 7.6)
“Lung Cancer Screening Decision Support Tool” HealthDecision^®^ http://healthdecision.org 21	Primary care clinicians, pulmonologists, nurse practitioners, physician assistants can use it with their patients Web‐based program, electronic health record software, English/French	Defined as a DA: 7 of 7 criteria Lowers the risk of bias: 9 of 9 criteria Other screening issues: 4 of 4 criteria Other quality issues: 13 of 13 criteria (Does not report readability levels: Average readability determined = Grade level 8.2)

a Stands for International Patient Decision Aid Standards (IPDAS). These three DAs are part of the A to Z Inventory—Patient Decision Aids, The Ottawa Hospital Research Institute. An IPDAS checklist is available from decisionaid@ohri.ca to help users assess DA qualifying criteria, criteria to lower risk of making a biased decision, other general and screening quality criteria.

b Responses to some unmet criteria are “no” or “unknown.”

c Readability was measured using readability software (http://readable.io). This software provides grade‐level scores according to five standardized reading scales (Flesch‐Kincaid Grade Level, Gunning Fog Score, Coleman‐Liau Index, SMOG Index and Automated Readability Index) along with an average of the five scores. For scoring purposes, we retrieved the average score using 175‐210 words of text from each of three DAs.

## 2 METHODS

### 2.1 DA development

A DA‐based on Janis and Mann's conflict theory of decision making,^24^, ^25^ the International Patient Decision Aid Standards (IPDASi v4.0)^26^ and findings from a statewide survey of PCPs^27^—was developed to facilitate a SDM discussion between PCPs and patients. Unique features of the DA included values clarification in regards to lung cancer screening using a decisional balance sheet (concept developed by Janis and Mann^24^, ^25^; self‐determination) that measured the effect of the decision on both the patient and others (relational autonomy). Both of these features help determine a patient's decision preference. Because inadequate health literacy is common—particularly among the elderly, ethnic minorities and socially disadvantaged populations—plain‐language and low‐literacy DA development strategies were used to enhance understanding by adults across health literacy levels.^18^, ^28^

In 2003, an international, collaborative group was established for the sole purpose of developing standards for patient DAs. This guideline, the IPDASi (the fourth version of which was released in 2014), includes 44 standards and 3 broad categories of criteria: (i) qualifying criteria, (ii) certification criteria and (iii) quality criteria.^26^ The qualifying criteria category is considered definitional, and the criteria are essential for designation as a DA. The certification category includes criteria that enhance avoidance of risk of harmful bias. Lastly, the quality criteria are designated non‐essential but are known to enhance a DA.

Results from a statewide survey of PCPs about knowledge, attitudes and use of lung cancer screening also informed DA development.^27^ Among 101 physicians surveyed, knowledge gaps existed about approved guidelines and reimbursement. Major physician concerns included unnecessary procedures, radiation exposure and patient anxiety.^15^, ^27^ These three physician concerns, among others, were addressed in the DA.

Using a model development process, components of the decision‐balance portion of the DA were developed by the primary author, and consensus was reached with the help of an interdisciplinary research team (composed of the coauthors), three nationally known content experts in decision making and lung cancer screening, plus three laypersons.^29^ The DA incorporated IPDASi standards, including all 7 of 7 defining criteria, 8 of 9 criteria to reduce bias, 2 of 4 criteria related to screening and 10 of 13 quality criteria (see Table 1 for comparisons).

The DA developed for this study is an eight‐page, 5.5 inch–by–8.5 inch booklet entitled *Is Lung Cancer Screening for You?*, written in plain language at the fifth‐grade reading level measured using readability software (http://readable.io). This software provides grade‐level scores according to five standardized reading scales (Flesch‐Kincaid Grade Level, Gunning Fog Score, Coleman‐Liau Index, SMOG Index and Automated Readability Index) along with an average of the five scores.^30^ The first page of the DA states “Lung cancer screening is not for everyone. It may be for you.” That page is followed by two pages describing factors that increase the risk of lung cancer. A three‐item checklist asks patients to describe their age and smoking status. On a later page, “five facts about lung cancer screening” are presented that relate specifically to Medicare coverage, benefits of screening, disadvantages of screening and screening frequency recommendations.

An interactive decision‐balance exercise entitled “What's important to you?” was incorporated into the DA, giving patients a chance to weigh the pros and cons for themselves and with others and to help them discuss their values with their PCP. The exercise involved completing a four‐cell table, where the patients were asked to select among 14 statements that reflected how they felt about lung cancer screening. Additional options were available to allow patients to create their own statements. The 14 statements were based on concerns reported during the primary author's experience with participants of another lung cancer screening study and on the literature (see Table 2).^31^, ^32^

**Table 2:** Patient responses to value statements on the DA

Screening statement	Affirmative response by patient (N = 20) n (%)
Pros of screening	
If I have early lung cancer it may be curable	17 (85%)
My family may decide to get a lung cancer screening too	16 (80%)
I will know more about the health of my lungs	14 (70%)
If my doctor and I plan to screen my lungs, I will worry less about lung cancer	13 (65%)
My family will be happy that I am taking care of my health	13 (65%)
A screening may uncover other health problems	12 (60%)
My family will worry less about my health	10 (50%)
Cons of screening	
My family may worry that a lung cancer will be found	5 (25%)
I am worried about being exposed to radiation	4 (20%)
Friends and family may blame me for having smoked	4 (20%)
I may get unneeded tests or treatments if the screening results are unclear or wrong	4 (20%)
I am afraid that I will have a lung cancer that is not curable	4 (20%)
I am worried about feeling like an outcast for smoking	3 (15%)
Others close to me will suffer if I have a health problem	3 (15%)

The final page of the DA asked patients to rate, on a 1‐item scale of 1 to 10, the importance of having a lung cancer screening in the next 30 days. Information about resources for smoking cessation and lung cancer are listed on part of the final page and on the back cover. The pages have colourful photographs related to the written content, and picture clinic, non‐clinic and occupational settings; PCPs and individual patients and families; both genders; and a variety of racial backgrounds and smoking statuses. The smallest font size is 14, and there is substantial white space.

A prospective, one‐group, mixed‐method design was used to evaluate the DA implementation and a SDM strategy. The setting was an urban academic family health centre in the south‐eastern United States serving a large proportion of low‐income individuals.

### 2.2 Recruitment

For the purpose of this pilot feasibility study, the recruitment goal was 20 patient‐PCP dyads. A list of 485 patients between the ages of 55 and 77 who listed Medicare or Medicaid as their primary health insurance was collected from the health centre's electronic medical record system. Two hundred of these patients were randomly selected to receive a mailed invitation at their home address. Patients were offered the opportunity to opt out of a recruitment telephone call by sending an email or leaving a voicemail message for the study coordinator. Of the patients called by the study coordinator, 57 had a disconnected telephone number. The recruitment plan included calling the remaining 143 patients up to four times in an attempt to establish contact. The first 20 interested patients who met these criteria were scheduled for an appointment during one of the three pilot testing events held at the health centre.

During the recruitment telephone calls, the study coordinator determined eligibility. Eligibility criteria followed the Medicare guidelines, specifying that the patient be (i) a Medicare beneficiary; (ii) between 55 and 77 years old; (iii) without current symptoms of lung cancer; (iv) interested in learning more about lung cancer screening; (vi) a current smoker, or a former smoker who had quit within the past 15 years, with at least a 30 pack‐year smoking history; (vi) able to speak and read English; and (vii) without a personal history of lung cancer. Interestingly, all 20 high‐risk patients recruited for this study stated they were not familiar with lung cancer screening prior to our communication with them.

### 2.3 Study procedures

A few patients required transportation, the cost of which was included in the grant budget. Transportation was provided by a private local taxi service known to the research team. At study completion, patients received a $25 cash “thank you” gift.

An invitational email recruited four PCPs to participate as part of the planned 20 PCP‐patient dyad interactions. It was not the intent to match the patients with their usual PCPs during this feasibility study. Therefore, some patients engaged in a SDM discussion with a PCP who was not their usual physician.

Approval was obtained from the local university hospital's institutional review board affiliated with its school of medicine, and each patient gave informed consent to participate after having the opportunity to read the informed consent form and ask questions of the study staff before meeting with their assigned PCP. The testing times were scheduled at one location over a 6‐week period.

A 1‐hour PCP orientation was conducted immediately before each of the three pilot testing events. Approximately 1 week prior to the orientation, each PCP received a collection of printed materials that included two published research articles about lung cancer screening research and current screening guidelines, along with a one‐page document entitled “Providers’ Brief Shared Patient Decision‐Making Guide,” which took a patient‐question‐and‐PCP‐answer format (see Table 3). The in‐person orientation included a review of this information and the pilot testing process. PCPs received compensation based on a dual employment relationship (school of medicine plus school of public health) established for the purpose of this study. Each PCP received $250 for each 4‐hour commitment. Patients were scheduled for 30‐minute appointments. Upon arrival, after completing the informed consent process and a demographic form, patients were instructed to read and complete the interactive sections of the DA in the waiting area prior to meeting with their PCP.

**Table 3:** Shared decision‐making guide: sample questions and responses

Participant question	PCP response
Am I at high risk for lung cancer?	It depends on several factors. The recommendations state that if you are a current smoker between ages 55 and 77 and have smoked one or more packs of cigarettes a day for 30 y, you are at high risk. If you quit smoking 15 or more years ago, you are not considered at high risk. Let's review your age and smoking history to determine if you are at high risk for lung cancer
What is the test like?	The screening uses a radiologic test to take a series of pictures of your lungs. During the test, you lie flat on your back in a doughnut‐shaped machine for just a few minutes
Should I worry about the amount of radiation I'll get during the test?	The dose of radiation used with each screening test is very low, but we don't yet know the effect of repeated screenings over time. The radiation dose of one screening is equal to the amount of radiation you would get over 6 mo doing your normal activities
What if the test shows that I have lung cancer?	If the screening shows a suspicious nodule, I will refer you to a specialist who will help us determine if it is cancer. The next step may involve another radiologic test or a surgical biopsy. If it is not cancer, we will watch it closely. If it is cancer, I will refer you to a cancer specialist to help us make important treatment decisions
Is the test covered by my insurance?	As of February 2015, in the U. S. Medicare & Medicaid have agreed to cover lung cancer screenings. If you are eligible, there is no cost to you. Most private insurances do also
Do you think there are more benefits than risks?	That's a good question. Screening is most beneficial to those with a significant smoking history. I think it's important to weigh the benefit of finding lung cancer early with the chance of having to undergo unnecessary procedures because of abnormal results. Whether or not you decide to get a lung cancer screening, stopping smoking is the most powerful way for you to lower your chance of dying from lung cancer or other serious illnesses. Continuing to smoke can shorten your life span and has many, many risks

In a private room, the patient and PCP of each dyad reviewed every page of the DA together. A research team member was present as an observer and used a 15‐item fidelity checklist to monitor the process and the content of the interaction and to record the time span of visit. A short survey was completed by both the patient and the PCP separately immediately after the dyad interaction. Six items measured the acceptability of and satisfaction with the DA and the SDM strategy. In addition, to the 6 items, 2 additional items asked both responders to rate their perception of how long the interaction lasted (less than 5, 5‐10, 11‐15 and more than 15 minutes) and about their decision (patient: I definitely want to get screened, I prefer to make my final decision after thinking about my doctor's opinion, I prefer that my doctor makes the decision, I want more information about whether or not to get screened; PCP: I definitely want this patient to be screened, I want this patient to consider having a screening, I don't think the patient will have a screening at this time, the patient and I want more information about whether or not to get screened).

A brief, four‐question exit interview was conducted with each patient. The interviews, conducted by graduate students experienced in interviewing techniques, were audio‐recorded and transcribed verbatim. Questions included are as follows: (i) “In what way did the booklet help you to understand the pros and cons of lung cancer screening?” (ii) “In what way did the booklet help you discuss lung cancer screening with your doctor?” (iii) “Did you decide to have a lung cancer screening? How do feel about your decision regarding screening?” and (iv) “Is there anything that you would like to share about this experience that we didn't ask about?”

### 2.4 Data analysis process

Descriptive statistics were used to characterize the study sample, perceptions of acceptability and decision preferences. Data were analysed using statistical procedures in SPSS^®^ (version 24). The sample size precluded the use of inferential statistics. Using a thematic analysis method by Braun and Clarke,^33^ two members of the research team reviewed the transcripts. The two reviewers engaged in an iterative process of reading and analysing the transcriptions. The reviewers proposed themes and reread the transcripts to affirm that the themes reflected the patients’ experiences. The team collectively discussed the interpretations and then reconciled any differences until there was congruence.

## 3 RESULTS

All 20 patients were current cigarette smokers or former cigarette smokers who had quit within the past 15 years, and all had 30‐plus pack‐year smoking histories. The mean age of patients was 64.5 years (range: 56‐73 years). Most were female (n = 14, 70) and unmarried (n = 14, 70). Half were African American. Annual household income was less than $50 000 for the majority of patients (n = 18, 90%). Half rated their health as fair or poor (n = 10, 50%). Three patients brought family members who accompanied them during the visit with the PCP.

Overall, patients felt there were more pros than cons [to talking with a PCP about being screened for lung cancer (see Table 2)]. On the DA itself, in a section entitled “What's Important to You?”, a majority of patients selected the following “pro” values: “If I have an early stage lung cancer it may be curable,” and “A few of my family members may decide to get a lung cancer screening too.” The “cons” (pros vs cons) statements reflecting patients’ values were not selected by many participants.

In response to a scale on the DA, a majority of patients (n = 16, 80%) rated the importance of a decision about lung cancer screening as a 10 of 10 before and after the discussion with the PCP. The others (n = 4, 20%) indicated that they “preferred to make a final decision after discussing lung cancer screening with their usual PCP.” After the discussions with patients, the PCPs wanted the majority of patients (n = 17, 85%) to consider screening.

Overall, the DA was rated highly according to all 6 acceptability criteria (see Table 4). Patients felt the DA was easy to read, easy to use and helpful for making a decision and discussing their personal values with a PCP. All of the PCP responses were positive as well. For the most part, the time involved with using the DA (the first DA some had ever used)—measured by self‐report and direct observation (see Table 4)—was acceptable. The majority of patient‐PCP interactions (n = 14, 70%) took 10 minutes or less. The longest interaction took 17.2 minutes. It was determined that this particular patient required a longer SDM interaction because of poor vision (Table 5).

**Table 4:** Decision aid (DA) acceptability

Criteria	Patients (N = 20)	PCPs^a^ (N = 4)
The DA helped with decision making (strongly agree or agree)	20 (100%)	18 (90%)
The DA was easy to read	20 (100%)	16 (80%)
The DA was easy to use	20 (100%)	17 (85%)
The amount of time required to read and use the DA was acceptable	20 (100%)	18 (90%)
The DA helped us (dyad: PCP and patient) discuss personal values	19 (95%)	16 (80%)
The DA increased my satisfaction with visit	19 (95%)	17 (85%)

a The 4 PCPs rated the DA after each interaction with 20 patients. Therefore, there were 20 ratings for each patient and PCP.

**Table 5:** Time taken by shared decision‐making process during dyad interaction

Length of time (self‐reported) (min)	Patients perceptions (N = 20)	PCP perceptions (N = 20)
<5	3 (15%)	1 (5%)
5‐10	11 (55%)	11 (55%)
11‐15	6 (30%)	7 (35%)
>15	0	1 (5%)

Time for each dyad interaction was self‐reported by participants and confirmed by direct, in‐person observation. Mean observational time was 11.6 min (range: 6.22‐17.2 min).

All patients agreed to participate in a brief exit interview. Major themes included patients (i) wanting to know whether they had lung cancer, (ii) reporting that using the DA helped their conversation with the PCP and (iii) being concerned over how a lung cancer diagnosis would affect family members. The majority of patients wanted to proceed with screening and felt the DA helped them make that decision with the PCP. Several patients wanted to be screened immediately. A 64‐year‐old female participant stated:

It's a decision I have to make. No one else can make it for me. So, I feel confident that I will do the test, have the screening done, and find out what's going on; if I do have it, it will be detected at an early stage.

Several patients were concerned about having a family history of lung and other cancers. A 68‐year‐old female participant stated: “My mother died with lung cancer. My dad died of throat cancer. And if I have it, I want to catch it in an early stage.” Many patients discussed other comorbidities that they had. One patient (a 73‐year‐old man) stated:

[The DA] makes you understand that, especially as a smoker at my age, and knowing that I have asthma and COPD [chronic obstructive pulmonary disease], and I sleep with a CPAP [continuous positive airway pressure], that this is something that I really need to do for myself.

Another male patient, 66 years old, stated:

[The DA] made it easy. Many times, I have different things going on with me, I really do not know how to present my concerns to my physician, and I walk out of the room. Well, the DA made me feel more comfortable. I knew exactly why I was here, and to be honest, it cleared my mind of questions before I got in the room. I could not believe it was so helpful. I always talk too much, and I was able to get directly to the point.

Patients were concerned about how a diagnosis of lung cancer would affect family members. They did not want to burden their loved ones. One patient (a 62‐year‐old male) stated: “[The DA] asked if I was worried about being a burden on my family, and I am. And it made me look at that, so that I would, you know, take better health care of myself.” At the end of the study, several patients who chose to proceed with lung cancer screening did so out of a motivation that early detection increases the chances of survival.

## 4 DISCUSSION

The most interesting finding was that a high percentage of patients (n = 16, 80%) expressed the desire to be screened for lung cancer after interacting with the DA alone, even before the SDM discussion with the PCP. We perceive that this diverse sample of at‐risk patients represents a group of current smokers and ex‐smokers who worry about their health and the possibility of being diagnosed with lung cancer.^34^ This motivated group of patients needs to make an informed decision. Achieving this goal is the greatest challenge with patients who have varying levels of health literacy and intense emotions regarding the threat of lung cancer. We agree that individuals need to understand the best available medical evidence relevant to a screening decision.^15^ Well‐designed DAs that enhance health literacy will promote an understanding of complex health information and an interest in preventive behaviours.

This pilot study reaffirmed that DAs can play an important role in promoting shared decisions about cancer screenings with high‐risk patients and their providers. However, no DA is sufficient to guarantee that clinical decision making is a shared experience.^35^, ^36^ There is widespread agreement that SDM works best when it includes certain elements. First and foremost, good SDM requires that clinicians have access to detailed knowledge of the latest evidence and a means to share it with patients and their family members in a way that supports comprehension, deliberation and thoughtful decision making.

Incorporating patients’ values and preferences is becoming a more common practice in health care. These considerations are essential to implementing an SDM discussion and are becoming more important in health‐care reimbursement policy, as evidenced by the coverage memorandum issued by the CMS^2^ for LDCT lung cancer screening. In preference‐sensitive decisions, such as whether to undergo lung cancer screening, the PCP alone does not have sufficient information to make an optimal decision for the patient. Embracing SDM requires a culture change in which PCPs prioritize patients’ self‐determination and relational autonomy and develop the skills to elicit these patient responses.^17^

Understanding the barriers to the use of DAs and a SDM strategy in the clinic setting will help to enhance their integration. We assumed the greatest barrier to implementation would be the time commitment involved. This study (in which a majority of patient‐PCP interactions took 10 minutes or less), together with evidence from more than 100 randomized control trials, provides no indication that an additional or unacceptable time commitment is required to engage in SDM in a clinical practice setting.^35^, ^36^ In a recent Cochrane review, in a subgroup analysis of 105 studies involving over 31 000 participants, the median effect of DAs on length of consultation was 2.6 minutes longer than usual care (no DA).^14^ However, perceived time constraints are the most frequently cited barrier to proposed change in clinical settings. Findings from a recently completed national survey of 810 PCPs (430 medical doctors and 380 nurse practitioners in the U.S.) indicated that only 30% and 37%, respectively, would be likely to engage in an SDM discussion with a patient if the time commitment was greater than 8 minutes.^37^, ^38^ In this sense, implementing SDM may be no different from implementing any other practice improvement.^35^, ^36^ Perhaps more common barriers to implementing new screening recommendations are PCPs’ knowledge and availability of resources in clinical settings, which are necessary for PCPs to assess eligibility for screening and to accurately describe the risks and benefits of screening in a time‐efficient manner.

The results of this pilot study suggest that brief, plain‐language, culturally sensitive DAs and SDM strategy can be developed to educate and engage PCPs and their high‐risk patients about new guidelines, SDM and the use of DAs. Making effective, tested DAs more available would facilitate SDM and improve the overall clinic experience for PCPs and patients alike.

One of the greatest strengths of this study was the sample's socio‐economic characteristics. Patients were diverse in gender, racial background and educational level, and they fit the qualifying characteristics for Medicare reimbursement of lung cancer screening (Medicare billing code G0296). A second strength was that fidelity was carefully monitored, and implementation of the DA was timed by direct, in‐person observation.

The findings are limited by the one‐group design and sample size. Testing the DA with the patient's usual PCP would have provided a clearer picture of how the DA would work when integrated into the typical flow of the patient's care. A second limitation was that follow‐up was not conducted to determine whether the patients received a lung cancer screening or met with their usual PCP to discuss screening and/or obtain a referral. The decision to forego this follow‐up was based on our desire to avoid circumventing the discussion between patients and their PCPs. We encouraged each patient to take the DA to an appointment with his or her PCP to discuss scheduling a lung cancer screening.

## 5 CONCLUSION

Strong evidence exists that patients exposed to DAs feel more knowledgeable, better informed, more certain about their own values and engaged in a more active role in decision making about their health choices. Our findings suggest that using a brief, interactive, theory‐based DA written in plain language at the fifth‐grade reading level in a clinical setting is acceptable to diverse group of patients and PCPs. These pilot study results will be used to refine and enhance the use and delivery of the DA in clinical settings with a more varied PCP workforce (nurse practitioners and physician assistants in addition to medical doctors). The results will also help determine a sample size for a full‐powered study that further explores the integration of SDM strategies between PCPs and their high‐risk patients with diverse health literacy skills and their adherence to their chosen decisions.

### 5.1 Practice implications

Now that evidence supports the value of lung cancer screening with LDCT and it is a covered service by most private and public insurance plans in the U.S. for selected patients described as high‐risk, it is important that implementation processes proceed in a way to maximize benefits, minimize harms and enhance sustainability. Demand for this service will increase as awareness about lung cancer screening increases. Brief clinic‐based strategies, with acceptable readability levels,^37^ are essential. Effectively translating and sustaining the use of DAs and SDM in clinic settings will require careful attention to implementation approaches.^39^

## CONFLICT OF INTEREST

None.

## ETHICAL DETAILS

The work described has been carried out in accordance with the Code of Ethics of the World Medical Association (Declaration of Helsinki) for experiments involving humans and with the Uniform Requirements for manuscripts submitted to biomedical journals. Informed consent was obtained from all participants.
